# Some Notes on the Treatment of Hæmoptysis in Pulmonary Tuberculosis

**Published:** 1908-06

**Authors:** Newman Neild

**Affiliations:** Assistant Physician to the Bristol General Hospital


### SOME NOTES ON THE TREATMENT OF HÆMOPTYSIS IN PULMONARY TUBERCULOSIS.

BY

NEWMAN NEILD, M.B., M.R.C.P.,

*Assistant Physician to the Bristol General Hospital.*

---

HÆMOPTYSIS in pulmonary tuberculosis is more than a sign of a disease, in that it requires treatment on its own account over and above the treatment required for the disease of which it is a sign. It requires treatment on its own account because it may help in the spread of the disease in the lungs, because the loss of blood may seriously weaken the resistance of the patient, because it may cause death by loss of blood or by suffocation.

I do not intend in these few notes to cover the whole ground of the subject, but merely to speak of one or two points upon which many of the standard text-books are silent or dismiss with a passing note.

We may roughly divide cases of hæmoptysis into three groups, according to severity. Into moderate hemorrhage, where the blood is mixed with the sputum ; comparatively free hemorrhage, where blood is brought up alone and in quantity, but does not immediately threaten life ; and profuse hemorrhage, immediately threatening the life of the patient.

The first group is a very tempting one for discussion, but I shall confine myself to making one observation upon it. It is generally considered that even slight staining or streaking of the sputum with blood means that direct extension of disease has caused the rupture of a small blood-vessel, this rupture being due to the breaking down of degenerating tissues in which the vessel lies. That this is the more common cause I readily admit, but there is another source that is a lesser cause of anxiety, and a cause more frequent in sanatoriums than elsewhere. This form of hemorrhage arises from the granulations covering some healing surface. Healthy granulations in other parts of the body have a tendency to bleed upon the least provocation, both from the delicacy of the walls of the new blood vessels and the friability of the newly-formed supporting tissues, and it is not difficult to understand how coughing causes, in similar granulations in the lungs, the slight injury required. We all know of cases where everything has been progressing most favourably for some months, when suddenly a small and apparently trivial hæmoptysis has so distressed the patient as to cause a serious set-back in the patient's general condition, apart from any increase of disease in the lungs. The chief value of the recognition of this cause of hæmoptysis lies in the fact that we may, in these cases, give the patient a simple and satisfactory explanation of the cause of their disquieting symptom, no small boon in a disease where the mental condition has such a profound effect upon the physical.

I may mention in passing, the slight aching pain and the dull pain on coughing that is sometimes caused by the dragging of hardening fibrous tissue upon the pleura. A recognition of this cause, and its explanation to the patient, may often be the means of sparing him much brooding and depression. We must not allow a really harmless symptom to do the patient harm.

In those cases where there is comparatively free hemorrhage unmixed with sputum, but with no immediate danger to life, there are well recognised lines of treatment, or "text-book" routine, in regard to which I wish to draw attention to two points: the danger of morphia, and the importance of posture.

This form of hemorrhage is sometimes followed by a spread of the disease, but we must be careful not to jump to the conclusion that there has been such spread solely on the ground that the hemorrhage has been followed by a rise of temperature for some days; nor must we too hastily conclude, as an alternative, that an increase of the disease has been the cause of both hæmoptysis and temperature. A rise of temperature is often caused by the absorption of effused blood in other parts of the body, for example in the abdomen after injuries, or in the alimentary canal after gastric hemorrhage, and there is no improbability in the view that blood absorbed from the lungs causes a similar temporary rise in the temperature. Although it may often be difficult to distinguish at first a rise in temperature due to extension of disease, it is not difficult to distinguish it from a non-tuberculous broncho-pneumonia, a condition that may follow a hemorrhage.

The presence of blood in the bronchioles and alveoli is a source of danger quite apart from its interference with respiration. It is a foreign substance, it is an unresisting pabulum for micro-organisms, and it may carry bacilli to uninfected parts. But these obvious points are often forgotten. In the first treatment of this type of case the sheet anchor has been morphia. It soothes the fears of the patient, quietens the action of the heart, and lessens the reflex cough. Instead of a restless and terrified patient with palpitation and continual cough, with expectoration of bright fluid blood, we have a quiet and fairly calm individual with a moderate pulse, who coughs far less and brings up less blood. Those who have once seen this change are only too liable to use the drug in a case of less severity.

I can best explain my objection to the routine use of morphia by giving the outline of a case where the bad effect of morphia was strongly impressed upon me.

The patient was a young man who had lived hard and suffered from excesses of various kinds, during which he had, many years before, contracted syphilis. He came uuder my care for a slight hæmoptysis, which soon ceased under treatment. He had had, during the previous five years, some three or four attacks of profuse hemorrhage, that had not been followed by any remarkable disturbance of health. There were slight but definite signs of an old lesion at the right apex, and there were no signs of its activity beyond the hemorrhage.

Some twelve months later I saw him again, suffering from a moderate hæmoptysis, and at this time a tertiary ulceration appeared upon the penis.

He was vigorously treated with potassium iodide and mercury, and the ulceration healed rapidly and the hemorrhage stopped. During this attack the blood was seen with the laryngoscope to come by way of the right bronchus. There was no expectoration beyond the blood, and although several examinations were made, no tubercle bacilli were found, and there were no alterations in the physical signs. Some months later I was called to see him in an attack of hæmoptysis, in which he had already brought up some half-pint of blood, and was in great mental distress, which I was unable to alleviate. I gave him $\frac{1}{4}$ gr. hypodermic of morphia. At this time he turned over on to the left side, and the cough and expectoration of blood having greatly diminished, he went to sleep in an hour or so. Unfortunately the blood was not examined for bacilli at this time, and I may here mention that in my experience blood free from other expectoration in cases of phthisis gives a negative result far more often than a positive when examined for bacilli. After this attack there was considerable oscillation of temperature, and scattered tubercle appeared in the left lung at various points. Tubercle bacilli were then discovered in the sputum. He was sent to a sanatorium, where, in spite of the most able treatment, he did not gain ground. He had frequent attacks of hæmoptysis of a mild character, and had staining of the sputum for prolonged periods.

In the end he had a severe attack of hæmoptysis, for which he was given morphia. By some mischance he was allowed to lie on the right side in the dozing condition following the injection, and a few days afterwards he developed a tuberculous pneumonia upon that side, where previously there had been least disease, and from this pneumonia he died.

I have in my mind other cases where I suspect morphia of having helped in the extension of the disease.

But beyond interfering with the removal of tubercle-laden blood, there is another reason why morphia should be avoided if we can do so. It is probable that morphia lowers the resistance of the body against tubercle, and the note by Dr. John M. H. Munro at the end of this paper supports this view. A drug that lowers the activity of the phagocytes is not the drug one would choose to use at a time when there may be a wide dissemination of tubercle bacilli in the air passages.

In many of these cases of intermediate severity the advantages and disadvantages of morphia must be carefully weighed. If possible it should be avoided, but when it must be given, then at least care should be taken that the after treatment assists in the removal of the blood as rapidly as possible by the free administration of drugs which dilute and increase the sputum. The cough that is caused by the increase of the sputum is nothing like so forcible or distressing as that caused by the thickened secretion that follows the administration of morphia, a thickening that is further increased by the removal of fluid by the bowel, and by lessening the intake of fluids by the mouth, that is to say, by the text-book secondary treatment for this form of hæmoptysis. I doubt much whether the attempt to reduce the blood-pressure by these means compensates, by its effect upon the pressure, for the decreased fluidity of the sputum.

Another point in the treatment of this type of hæmoptysis is the posture of the patient. The patient should be placed in a position as nearly horizontal as the necessity for expectorating will allow, but, above all, the patient should lie upon the side where the physical signs have proclaimed the greatest destruction of lung tissue. An argument frequently raised against the adoption of this position is that the hemorrhage does not necessarily come from the lung where there is most disease. This argument shows ignorance of the reason for which this position is adopted. It is not adopted in order that the effused blood shall remain in the lung where it arises, but lest any unexpectorated blood should remain in the lung where there is the greater amount of healthy lung tissue. Where there is more damaged lung there is less opportunity of doing harm to healthy lung.

The third group is a comparatively uncommon one, and it is still more uncommon for us to have the opportunity of treating it. It has been frequently stated, indeed I have but recently seen it clothed in all the impressiveness of the aphorism, that patients do not drown from hæmoptysis. I should not trouble to disturb a merely theoretical heresy, but this theory has a dangerous bearing upon practical treatment. To confine one's attention to preventing the patient's death from loss of blood, whilst the patient is allowed to die from suffocation beforehand, is very one-eyed treatment. I have been actually present in only two of these cases, although I have come in shortly after the heart had ceased to beat in others, and in some of these cases the *post-mortem* conditions have convinced me that death was due to obstruction of the respiratory tract. I have seen a case where death was due to hemorrhage into enormous excavations in the lung, without there having been any expectoration of blood, the affected parts being entirely insensitive, and the source of the hemorrhage being also within this area, and I admit that there are many cases in which death is due directly to loss of blood, and this in a very short time.

In my experience of the third group, the patient sits up in an attempt to breathe more freely, and in so doing assists the process of drowning. The blood may be blown out with a gurgling, choking sound rather than coughed up, and the patient struggles in his attempt to obtain air, the blood rapidly fills the lower tubes and is aspirated into the alveoli, so that there is soon an insufficiency of air behind the blood to drive the blood out. This is obviously no time for prescribing morphia and ice to suck! The patient must be placed at once on a bed or couch, turned upon the face with the head and shoulders hanging well down over the side, so that the tracheal and bronchial drainage may be as free as possible. Amyl nitrite should also be given as an inhalation.

More vigorous treatment is essential where the patient has already been allowed to fill up his respiratory tubes with blood, and the respiratory movements have ceased or are ineffectual. The risk of increasing the hemorrhage is of small moment compared with the absolute necessity of freeing the trachea and lungs from effused blood. The position described should be adopted at once, or the patient actually inverted, and if the blood does not at once pour from the mouth, the chest should be forcibly compressed, and every endeavour made to free the air passages of clots, and then artificial respiration resorted to until natural breathing is restored or the heart has irrevocably stopped.

Where a patient, in previous attacks of less severity, has been rightly treated with elaborate precautions against the least unnecessary movement, and when even the slightest percussion was tabooed, it requires courage to treat the patient energetically in a hæmoptysis of the utmost severity.

We may at times save a patient from drowning only to see him die from loss of blood, in which case it may be difficult to persuade the friends that although our vigorous treatment may have assisted the death from hemorrhage, the patient would certainly have died from suffocation if we had treated him otherwise; in short, it may be difficult to convince the friends that we have given the patient his only chance.

NOTE.—Dr. John M. H. Munro has most kindly allowed me to add the following notes of some results of his research upon the influence of various drugs upon phagocytosis:—

At the November meeting of the Bristol Medical Research Club, in the course of a preliminary communication on the "Influence of Drugs on Phagocytosis," the following experiments with morphia were cited. Unweighed traces of morphia were dissolved in normal serum, the opsonic index of which was then determined in comparison with that of the same normal serum to which no morphia had been added. Using an emulsion of tubercle bacilli and making full counts of 100 leucocytes, the addition of morphia to the serum on five different dates reduced the number of bacilli ingested from 97 to 72, 118 to 52, 94 to 52, 68 to 29, and 77 to 52, thus reducing the opsonic index on the average to 0.56. Similar results are obtained when serum diluted with morphinated normal saline is tested against the same serum diluted to the same extent with pure normal saline. In the case of some other drugs, the index is very noticeably reduced when the amount added is under 0.25 per cent. of the serum, but in the case of morphia the results have not yet been accurately quantified. The action of the morphia is presumably on the leucocytes, but this requires proof.

